# Plasma CA125 as a Prognostic Marker in Very Elderly Patients Hospitalized for Acute Heart Failure

**DOI:** 10.3390/jcm15062156

**Published:** 2026-03-12

**Authors:** Javier Jaramillo-Hidalgo, Mónica Ramos, Maribel Quezada-Feijoó, Rocío Toro, Noemí García-Calderón, Francisco Javier Gómez-Pavón

**Affiliations:** 1 Geriatrics Department, Hospital Central de la Cruz Roja, C/Reina Victoria, 24, 28003 Madrid, Spain; 2 School of Medicine, Universidad Alfonso X el Sabio University, Avda. de la Universidad, 1, Villanueva de la Cañada, 28691 Madrid, Spain; monica.ramos81@gmail.com (M.R.);; 3 Cardiology Department, Hospital Central de la Cruz Roja, C/Reina Victoria, 24, 28003 Madrid, Spain; 4 Biomedical Research and Innovation Institute of Cadiz (INiBICA), 11009 Cadiz, Spain; 5 Medicine Department, School of Medicine, Cádiz University, Edificio Andrés Segovia 3ª Floor, C/Dr Marañón S/N, 21001 Cadiz, Spain

**Keywords:** acute heart failure, elderly, CA125, NT-proBNP, prognosis, mortality, hospital readmissions

## Abstract

**Background/Objectives:** Acute heart failure (AHF) is a leading cause of hospitalization and mortality among very old patients, yet this group is underrepresented in prognostic studies. Carbohydrate antigen 125 (CA125) has emerged as a potential biomarker of congestion and inflammation, but its value in patients aged 80 years and over remains unclear. We aimed to evaluate the prognostic value of plasma CA125 measured at admission for 12-month all-cause mortality and the composite outcome of mortality or heart failure (HF) readmission in very elderly patients hospitalized for AHF. **Methods:** We conducted a prospective observational study of patients aged ≥80 years admitted to an acute geriatric unit for AHF. CA125 and NT-proBNP were measured within 24 h of admission. Outcomes were assessed at 12 months. Survival analyses were performed using Kaplan–Meier curves, Cox regression models, and restricted cubic splines. **Results:** A total of 210 patients (mean age 89.8 ± 5.3 years; 75.3% females; 88.1% frail) were recruited. During the one-year follow-up, 70 deaths (37.2%) and 68 HF hospital readmissions (36.1%) were recorded. Patients in the highest CA125 tertile had an increased cumulative mortality risk (log-rank *p* = 0.061). A CA125 value ≥ 100 U/mL independently predicted both mortality (HR 1.88, 95% CI 1.15–3.09; *p* = 0.012) and the composite endpoint (HR 1.54, 95% CI 1.04–2.29; *p* = 0.031). Measures of functional dependence and frailty demonstrated greater discriminative ability than biomarkers. **Conclusions:** In very elderly patients hospitalized for AHF, elevated CA125 at admission independently predicted 12-month mortality and HF readmission. CA125 provides complementary prognostic information to geriatric assessment and may support risk stratification in this vulnerable population.

## 1. Introduction

Heart failure (HF) is a major public health problem in developed countries, with prevalence and incidence increasing markedly with age. Among patients aged 80 years and older, HF represents one of the leading causes of hospitalization and mortality. Despite its clinical relevance, this population remains underrepresented in most clinical trials and observational studies, especially those including patients with advanced frailty. Consequently, the applicability of existing evidence to routine geriatric practice is limited [1,2,3,4,5,6].

Older adults with HF differ substantially from younger cohorts. They typically present with a higher burden of comorbidities, polypharmacy, functional dependency, and frailty, which not only modify the clinical expression of HF but also influence prognosis and response to conventional therapies. In this setting, tools to improve risk stratification and support individualized management are urgently needed [6,7,8].

Biomarkers play a central role in HF diagnosis and prognosis. Natriuretic peptides are widely used, but their interpretation in very old patients is often challenging due to age-related physiological changes, renal dysfunction, and multimorbidity. In recent years, carbohydrate antigen 125 (CA125), a glycoprotein traditionally employed as a tumor marker in ovarian cancer, lung cancer, teratoma, and non-Hodgkin lymphoma, has emerged as a potential biomarker in HF. CA125 levels rise in response to serosal inflammation and systemic congestion, both hallmarks of acute heart failure (AHF). Several studies have demonstrated associations between elevated CA125 and congestion severity, longer hospital stays, and worse outcomes, including mortality and hospital readmissions. Moreover, CA125 is inexpensive, widely available, and less influenced by renal function than natriuretic peptides [9,10,11,12,13,14].

However, most available evidence derives from middle-aged or younger-old patients, with a mean age in the early seventies. Data focusing especially on the old-old (≥85 years) remains scarce. Furthermore, the interplay between CA125 levels and geriatric features such as frailty, functional status, and multimorbidity has been insufficiently investigated, despite their well-established impact on HF prognosis [6,7,8,15,16,17].

To address this gap, we have conducted a prospective observational study in patients aged 80 years and older admitted with AHF. We hypothesized that elevated CA125 levels at admission would be independently associated with a higher risk of all-cause mortality and the combined endpoint of mortality or HF hospital readmissions at one-year follow-up. In addition, we aimed to compare the prognostic performance of CA125 with established biomarkers and geriatric variables related to frailty and functional status.

## 2. Materials and Methods

### 2.1. Study Design and Setting

We conducted a prospective, single-center observational study designed to evaluate the prognostic value of CA125 in very elderly patients hospitalized for AHF. Consecutive patients were recruited between 9 March 2023 and 1 March 2024 in the acute geriatric unit of Hospital Universitario Central de la Cruz Roja, Madrid (Spain). All patients were followed for 12 months after hospital discharge.

### 2.2. Study Population

Eligible participants were consecutive patients aged ≥80 years admitted with a primary diagnosis of AHF, as defined by the 2021 European Society of Cardiology criteria [1]. Exclusion criteria were age less than 80 years, lack of informed consent, or active malignancy.

### 2.3. Sample Size

The required sample size was calculated to detect a statistically significant hazard ratio (HR) between CA125 and time to mortality during follow-up. According to previous studies [18], an estimated HR of 1.78 for all-cause mortality and 1.58 for the composite outcome of mortality or AHF hospitalization was assumed. To obtain a conservative estimate, the lower HR (1.58) was used.

For a confidence interval (CI) 95% and 80% statistical power, a minimum sample size of 166 patients was required. Assuming a 20% follow-up loss rate, the final target sample size was 208 patients, rounded up to 210 participants.

### 2.4. Clinical Assessment and Data Collection

Within the first 24 h of admission, standardized data collection was performed. Sociodemographic variables included age, sex, and living situation (home or nursing facility). The burden of comorbidities was assessed using the Charlson comorbidity index [19], and the presence of hypertension, diabetes mellitus, chronic kidney disease, atrial fibrillation, and coronary artery disease was recorded. A comprehensive geriatric assessment (CGA) was conducted in all patients, including: (i) frailty using the clinical frailty scale (CFS) [20], (ii) the functional status through the Barthel index (BI) [21], (iii) a nutritional evaluation using mini-nutritional assessment short form, and (iv) polypharmacy defined as the use of ≥5 medications.

The physical examination focused on signs of congestion, including jugular venous distension, peripheral edema, hepatomegaly, and orthopnea. The imaging and cardiac evaluation were performed with chest radiography, a 12-lead ECG, and a bedside ultrasound, using the Philips Lumify S4-1 (Philips Ultrasound, Bothell, WA, USA) to assess the inferior vena cava diameter and the presence/absence of a pleural effusion. Additionally, a transthoracic echocardiogram was performed during hospitalization if no examination had been performed within the previous 6 months (Philips Affinity-70C model, Philips Ultrasound, Bothell, WA, USA), to assess the left ventricular function, considering reduced ejection fraction when it was <50% [1], and to identify significant pulmonary hypertension when present [22].

Routine laboratory testing included basic hemogram and biochemical variables, including hemoglobin, creatinine, estimated glomerular filtration rate (CKD-EPI), sodium, and potassium.

In the case of a patient with chronic HF therapy, this information was documented at the time of admission.

### 2.5. Biomarkers Measurement

Plasma concentrations of CA125 and NT-proBNP were determined within 24 h of admission. Blood samples were obtained under standardized conditions and processed according to the hospital laboratory protocols. CA125 concentrations were determined using a luminescence-based immunometric immunoassay (Vitros CA125; Ortho Clinical Diagnostics, Madrid, Spain). NT-proBNP levels were measured by a fluorescence immunoassay (VIDAS NT-proBNP2; bioMérieux, Marcy-l’Etoile, France). The results were expressed in U/mL for CA125 and pg/mL for NT-proBNP.

For statistical analyses, CA125 was analyzed both as a continuous variable and in tertiles. Additionally, a predefined cut-off point was used (CA125 > 100 U/mL). To select this, different cut-off points were explored for the study event, and we chose the one that yielded the highest odds ratio (OR) and statistical significance in preliminary analyses at 6 months.

### 2.6. Outcomes

The primary endpoint was all-cause mortality within 12 months after hospital discharge. The secondary outcome was a composite of all-cause mortality or HF hospital readmissions within 12 months. In-hospital mortality was recorded but excluded from the survival analyses. Heart failure–related readmissions were defined as unplanned hospitalizations with heart failure as the primary discharge diagnosis from hospital administrative records. For analytical purposes, only the first readmission occurring during the follow-up period was considered. Follow-up data were obtained through review of electronic medical records and, when necessary, by telephone contact with patients or caregivers.

### 2.7. Statistical Analysis

Continuous variables are presented as mean ± standard deviation or median (interquartile range), according to data distribution. Categorical variables are expressed as counts and percentages. Group comparisons were performed using Student’s *t*-test or the Mann–Whitney U-test for continuous variables, and the χ^2^ test or Fisher’s exact test for categorical variables.

Time-to-event outcomes were analyzed using Kaplan–Meier survival curves, with log-rank comparisons performed. Follow-up time was calculated from hospital discharge to the event of interest or censoring at 12 months. Patients who died during the index admission were excluded from time-to-event analyses. Associations between CA125 levels and outcomes were evaluated using Cox proportional hazards regression models, and results are reported as hazard ratios (HRs) with 95% confidence intervals (CIs). The relationship between continuous CA125 levels and outcomes was further explored using restricted cubic spline regression. Prognostic discrimination was assessed using receiver operating characteristic (ROC) curves.

All statistical tests were two-sided, and *p*-values < 0.05 were considered statistically significant. Analyses were performed using SPSS version 28.0 (IBM Corp., Armonk, NY, USA) and Stata version 17.0 (StataCorp, College Station, TX, USA).

### 2.8. Ethical Considerations

The study protocol was reviewed and approved by the Institutional Ethics Committee of Universidad Alfonso X el Sabio (approval no. 2023_03/179). Written informed consent was obtained from all patients or their legal representatives. The study was conducted in accordance with the Declaration of Helsinki.

## 3. Results

### 3.1. Baseline Characteristics

A total of 210 patients aged 80 years and over were enrolled in the study. The mean age of the subjects was 89.8 ± 5.3 years, and 75.3% were females. Frailty was highly prevalent, with 88.1% of patients presenting a CFS score ≥ 5, and 37.6% showing moderate-to-severe functional dependence (BI < 60). HF with preserved ejection fraction (HFpEF) was the predominant phenotype (80.2%). In-hospital mortality occurred in 22 patients (10.5%), who were excluded from time-to-event analyses. Baseline clinical, functional, and laboratory characteristics are summarized in Table 1.

Median CA125 concentration at admission was 43 U/mL (IQR 22.3–96.6), and median NT-proBNP was 5424 pg/mL (IQR 2922–11,034).

### 3.2. Primary and Secondary Outcomes

The prognostic analyses were restricted to 188 patients discharged alive, after the index admission during which recruitment was performed, and followed for 12 months. Over a period of one year, 70 deaths (37.2%) were recorded, and 66 patients (36.1%) experienced at least one hospital readmission due to HF.

The baseline characteristics according to CA125 tertiles are shown in Table 2**.** Patients in the highest CA125 tertile did not differ significantly from those in the lower tertiles with respect to age, sex, living situation, degree of functional dependence, frailty status, or comorbidity burden. However, a trend towards higher atrial fibrillation prevalence was observed across increasing CA125 tertiles. A progressive increase in mortality was observed across the CA125 tertiles, with the highest tertile showing a higher one-year mortality rate than the lower two tertiles, with a trend toward statistical significance (*p* = 0.08).

**Figure 1:**
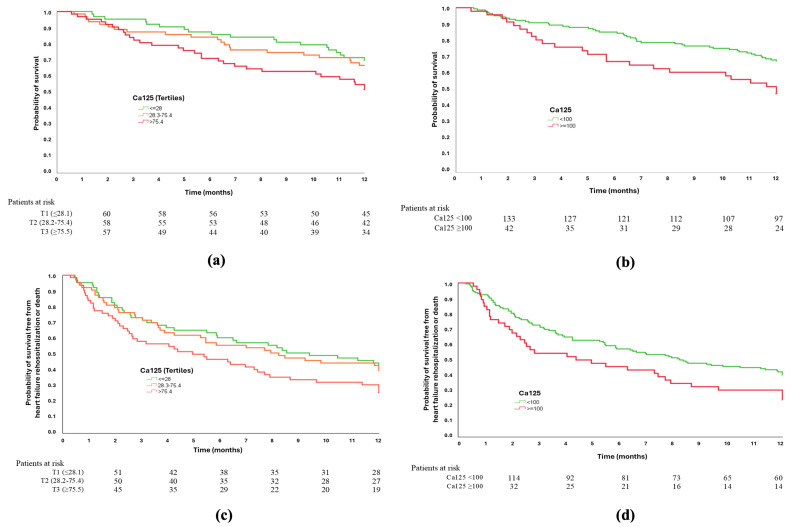
Kaplan–Meier curves showing time-to-event outcomes during follow-up. (**a**) one-year mortality according to CA125 tertiles; (**b**) One-year mortality according to a CA125 cut of 100 U/mL; (**c**) combined endpoint according to CA125 tertiles; (**d**) combined endpoint according to a CA125 cut-off of 100 U/mL. Curves represent the cumulative probability of events over time according to study groups. Abbreviations: CA125: carbohydrate antigen 125.

We examined the association between admission CA125 and all-cause mortality. When patients were stratified into three groups based on their CA125 tertiles, the Kaplan–Meier curves demonstrated a progressive separation, with a higher cumulative mortality observed in the top tertile (log-rank *p* = 0.061) (Figure 1a). When the pre-specified threshold of 100 U/mL was applied, survival differences became more evident: individuals with CA125 concentrations of 100 U/mL and over had significantly lower 12-month survival rates compared to those with lower concentrations (log-rank *p* = 0.011) (Figure 1b). In Cox univariate models, CA125 in the highest tertile (vs. the lowest) was associated with an almost twofold increase in the hazard of death (HR 1.89, 95% CI 1.06–3.35; *p* = 0.031). Dichotomization at 100 U/mL yielded a similar effect size (HR 1.88, 95% CI 1.15–3.09; *p* = 0.012), supporting the clinical relevance of this pragmatic cut-off (Table 3).

We then evaluated the composite of all-cause mortality or HF rehospitalization at 12 months. The Kaplan–Meier analysis once again revealed a graded risk across the CA125 tertiles, with a tendency towards higher event rates in the upper tertile (log-rank *p* = 0.069) (Figure 1c). The 100 U/mL threshold identified a subgroup with clearly higher cumulative risk for the combined endpoint (log-rank *p* = 0.029) (Figure 1d). Consistently, Cox univariate regression demonstrated increased hazards for patients in the top tertile (HR 1.60, 95% CI 1.03–2.48; *p* = 0.035) and for those with CA125 ≥ 100 U/mL (HR 1.54, 95% CI 1.04–2.29; *p* = 0.031) (Table 3). Next, to characterize the stability of these associations across parameterizations, we compared tertile-based and cut-off-based analyses. In addition, multivariable Cox regression analyses further adjusted for geriatric assessment variables—including the Barthel Index, Charlson Comorbidity Index, and MNA-SF, selected to represent key domains of geriatric vulnerability (functional status, comorbidity burden, and nutritional status)—the association between CA125 and the study outcomes remained consistent in both magnitude and direction. Effect estimates were not substantially attenuated after this adjustment, supporting the independent prognostic value of CA125 beyond these clinically relevant markers of frailty and overall health status.

**Figure 2:**
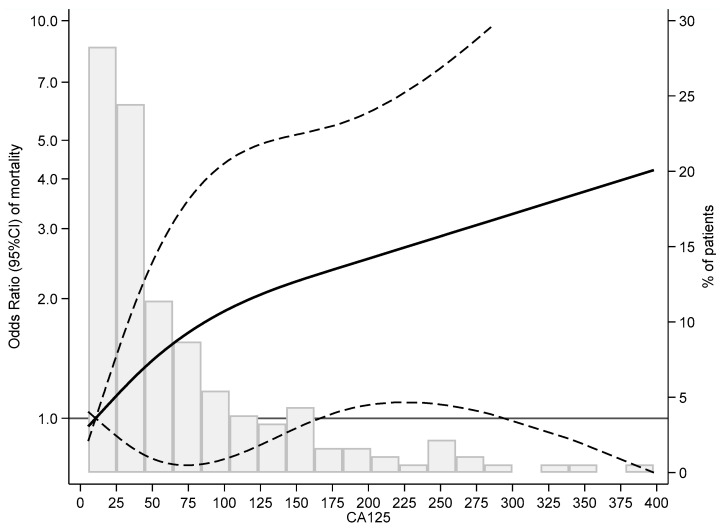
Cubic spline analysis of 12-month mortality by CA125 level. The x-axis shows CA125 levels (U/mL). The left y-axis represents the odds ratio (OR) for mortality with 95% confidence intervals (solid and dashed lines). The right y-axis shows the percentage distribution of patients across CA125 values (gray histogram). Abbreviations: CA125: carbohydrate antigen 125.

We performed a logistic regression using cubic splines. This revealed a direct, increasing, and almost linear relationship between CA125 and one-year mortality. There was a rapid increase up to CA125 values of 100 U/mL, followed by a less pronounced rise at higher levels. Moreover, the association reached statistical significance only at very high values, specifically above 150 U/mL (Figure 2).

**Figure 3:**
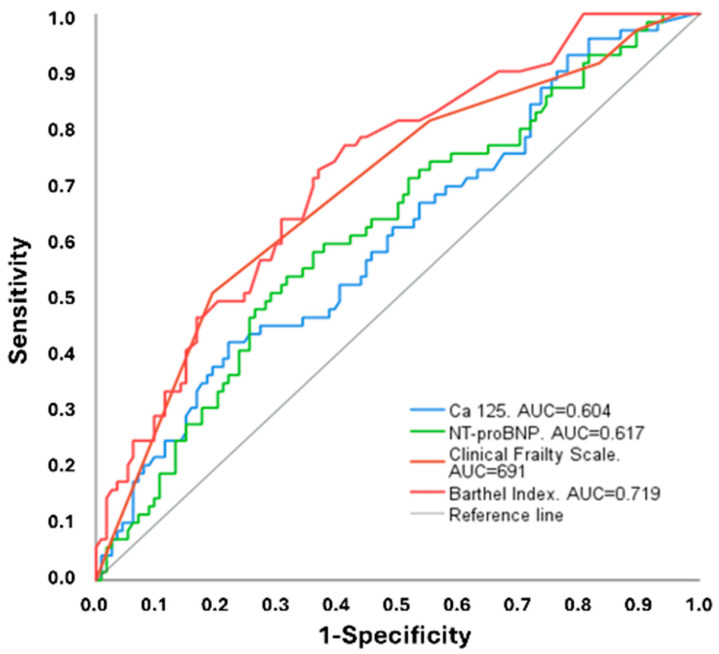
ROC curves for 12-month mortality using CA125, NT-ProBNP, clinical frailty scale, and Barthel index. Abbreviations: AUC: area under the curve; CA125: carbohydrate antigen 125; NT-proBNP: N-terminal pro-B-type natriuretic peptide.

When comparing prognostic discrimination, ROC curve analysis highlighted the superior performance of geriatric variables over biomarkers. The BI showed the strongest predictive capacity for one-year mortality (AUC 0.719, 95% CI 0.645–0.794), followed by the CFS (AUC 0.691, 95% CI 0.611–0.770). In contrast, NT-proBNP (AUC 0.617, 95% CI 0.533–0.701) and CA125 (AUC 0.604, 95% CI 0.520–0.689) displayed lower discriminative power (Figure 3). These findings suggest that functional dependence and frailty, as measured by the BI and CFS, were more effective predictors of mortality than the cardiac biomarkers in this very elderly cohort.

## 4. Discussion

This study, conducted in a cohort of hospitalized seniors with AHF, shows that CA125 measured at admission is a consistent prognostic marker for 12-month all-cause mortality and for the composite outcome of mortality or HF hospital readmissions. Our results, for the very first time, demonstrate the usefulness of this biomarker for early risk stratification in a population with a mean age close to 90 years. This population is typically belittled in AHF research studies but is exposed to the highest burden of adverse outcomes.

Our findings contribute to a growing body of evidence recognizing CA125, traditionally considered an oncological sign, as a marker of systemic congestion [18,23,24,25,26] and serosal inflammation with prognostic implications in HF. Prior studies, mostly in AHF but also in chronic stable HF [14], have shown that elevated CA125 levels correlate with congestion severity and increased risk of mortality and rehospitalization [27,28,29,30,31,32,33,34,35]. It is worth highlighting a systematic review published by Li et al. [31], which demonstrated that elevated CA125 levels were associated with a 68% increase in all-cause mortality (HR 1.68, 95% CI 1.36–2.07; *p* < 0.0001; I^2^ = 74%) and a 77% increase in HF–related readmissions (HR 1.77, 95% CI 1.22–2.59; *p* < 0.01; I^2^ = 73%). A more recently published systematic review also revealed CA125 as a significant prognostic factor for mortality and hospitalization risks in the acute and chronic HF population [36]. In 2025, Garcia et al. [37] confirmed that high C-reactive protein (CRP) levels, when coexisting with high CA125 levels, were associated with a higher mortality risk in a cohort of AHF with preserved EF. Concurrently, Miñana et al. [34], in a large cohort of patients with HFpEF, concluded that CA125 predicted the long-term burden of total HF admissions and was associated with the risk of long-term all-cause mortality. Agreeing with these authors, NT-ProBNP shows a lack of predictivity in AHF readmission due to the involvement of right HF, renal dysfunction, elderly patients, critical illness, and obesity; but also causes that provoke myocardial end-diastolic stress, such as pulmonary embolism or pneumonia. As proposed by the European Society of Cardiology, this marker needs to be supported by dyspnea and interpreted as a continuous variable [9]. The key contribution of our study is to confirm CA125’s prognostic value in an extremely old population characterized by advanced frailty, multimorbidity, and functional dependence.

A particularly practical finding is the identification of a clinically meaningful threshold. While some previous studies have focused on low CA125 levels to identify low-risk patients (e.g., <23 U/mL in the series by Núñez et al. [11]. In our cohort, elevated values (≥100 U/mL) identified a subgroup at substantially higher risk of both mortality and the composite endpoint at 12 months. These results highlight the potential usefulness of CA125 as a simple, widely accessible tool for prioritizing follow-up and the intensity of post-discharge care. Despite these findings, the proposed cutoff should be interpreted cautiously, given that it was derived from exploratory analyses within the same cohort and has not yet been validated in an independent population. Exploratory modelling using splines yielded a similar dose–response pattern, reinforcing the internal consistency of the association.

The composite endpoint of HF rehospitalization or mortality is particularly relevant in the geriatric scenario, as it reflects the care burden and morbidity that condition quality of life and resource use. In our cohort, elevated CA125 was significantly associated with increased risk of the composite at 12 months, consistent with previous large studies reporting that higher concentrations of the biomarker are accompanied by a progressive increase in the risk of death or HF hospitalization [18,30,31,34,38,39]. Our results are consistent with those reported by Nuñez et al. [18] in a cohort of 2356 patients hospitalized for AHF, where increasing CA125 quartiles independently predicted the combined outcome, with the highest risk observed in the upper quartile (HR = 1.72, 95% CI: 1.36–2.20; *p* < 0.001). Similar associations have been described by Miñana et al. [34], who observed a non-linear relationship between higher CA125 levels and HF readmission in 2369 patients with HFpEF. Also, Li et al. [31] reported an increased risk of HF hospitalization in patients with elevated CA125 levels (HR = 1.51, 95% CI: 1.11–2.04; *p* < 0.01). However, Llácer et al. [35] reported a weaker and non-significant association in a very elderly HFpEF cohort, more comparable to ours. This aligns with our preliminary 6-month analysis, in which CA125 did not significantly predict HF readmission, although a trend was observed in the highest tertile. Taken together, these data suggest that in older adults with HFpEF, the prognostic performance of CA125 may be attenuated when hospitalization is analyzed as an isolated outcome, rather than as part of a composite endpoint.

CA125 showed modest ability to predict mortality in our cohort, with performance notably lower than previously reported in larger studies, as Chen et al. reported (AUC: 0.845 for CA125) [33]. This may reflect the smaller sample size, the advanced age, the comorbidity burden, and the functional impairment of our population. By contrast, measures of functional dependence, using BI, and frailty demonstrated stronger discriminative value. These data may suggest that geriatric vulnerability overestimates biomarker-based risk in the old-old adults hospitalized for AHF. This observation is consistent with previous publications showing that higher BI scores at discharge were associated with lower mortality and reduced readmission risk, whereas poor functional status was associated with worse outcomes [16,40,41]. Frailty has also been repeatedly shown to predict adverse outcomes, independent of age and left ventricular function. Yuguchi et al. [42] recently reported a significant association between higher CFS scores and increased mortality risk (HR: 3.39, 95% CI: 1.32–8.72; *p* = 0.011), with an AUC of 0.74 for mortality prediction. Sunaga et al. [15] reported similar findings (HR: 2.54, 95% CI: 1.39–4.66; *p* = 0.003). The combination of frailty with elevated CA 125 appears to define the subgroup with the poorest prognosis, whereas individuals with preserved physical function and low CA125 demonstrated comparatively favorable outcomes [35,43]. This supports a dual approach to risk stratification—integrating biomarkers reflecting congestion and systemic inflammation with measures of functional capacity and frailty. Based on our results and the recommendations proposed by Jiménez-Méndez et al. [44], an optimal risk-stratification approach in the senior population may combine functional and frailty scales with blood-based biomarkers, such as CA125, to identify patients at high risk who may benefit from intensified management and closer follow-up.

Compared with previous evidence, our study broadens the field of application of the biomarker: it confirms that prognostic value is preserved when analyzing very elderly patients, with predominant HFpEF and high multimorbidity, and when using a high threshold (≥75–100 U/mL) that concentrates the most clinically relevant risk in this profile. In contrast to studies proposing low thresholds to define low risk [11,35], our data provides the necessary counterpart for practice: a high cut-off, operational for detecting high risk and guiding follow-up and treatment decisions.

In clinical application terms, the most directly transferable finding is that measuring CA125 at admission allows classification of elderly AHF patients into risk levels and identification of those with a substantially higher likelihood of dying or being re-hospitalized within one year. More recent studies have further suggested that serial measurement of this biomarker at discharge (in patients with a length of hospital stay >10 days) may have prognostic implications in terms of mortality [45]. This information can help prioritize resources (discharge planning, education, telemonitoring), adjust therapeutic thresholds (e.g., diuresis intensity with close monitoring of renal function), and structure follow-up (early visits, coordination with primary care). In this context, clinical trials have been published demonstrating the usefulness of CA-125–guided diuretic treatment strategy compared with standard management [46]. In addition, given its low cost, its use could be generalized without requiring additional infrastructure.

Several limitations materially constrain the interpretation of CA125 in this study. First, this was a single-center observational cohort with a relatively small sample size, which may limit statistical power and the generalizability of the findings. Furthermore, residual confounding cannot be excluded, and the external validity of the results should therefore be interpreted cautiously. Second, the modest sample size substantially reduced statistical power—particularly for interaction analyses—raising the likelihood of false-negative findings. Third, echocardiographic evaluation was not standardized, and key parameters (including pulmonary pressures and right ventricular function) were not systematically collected. Fourth, biomarkers were obtained within 48 h of admission; although acceptable for CA125 given its long half-life, this sampling window is likely suboptimal for NT-proBNP, which may be more informative when measured closer to discharge. Fifth, post-discharge CA125 trajectories were not captured, precluding assessment of dynamic risk stratification and longitudinal response. Sixth, the CA 125 threshold of 100 U/mL was derived from exploratory analyses within the same study cohort and was not externally validated, which may limit its generalizability and should be confirmed in independent populations. Finally, these results pertain to acute heart failure and should not be generalized to stable heart failure populations.

The study also has notable strengths: we studied one of the oldest cohorts reported (predominantly nonagenarians), captured an exhaustive CGA (frailty, functional status, and geriatric syndromes), achieved high data completeness, and completed 12-month follow-up despite logistical challenges. These features enhance real-world relevance and underscore the value of geriatric–cardiology collaboration for risk stratification in very old patients with AHF.

## 5. Conclusions

In very elderly patients hospitalized for AHF, CA125 measured at admission was independently associated with 12-month mortality and with the composite of mortality or HF rehospitalization. A pragmatic threshold of ≥100 U/mL identified a high-risk subgroup with clearly worse outcomes. CA125 is simple, inexpensive, and widely available, and provides complementary prognostic information to cardiogeriatric assessment. Routine incorporation of CA125 into the evaluation of very old patients with AHF may support individualized care planning and prioritization of post-discharge follow-up. Further multicenter studies should evaluate whether CA125-guided strategies can improve outcomes in this highly vulnerable population.

## Figures and Tables

**Table 1:** Baseline characteristics.

Variable ^a^	
N	210
Age, years, mean (SD)	89.8 (5.3)
Female sex, n (%)	158 (75.2)
BMI, kg/m^2^, mean (SD)	25.5 (5.2)
Living situation, n (%)	
Home	129 (61.4)
Nursing home	81 (38.6)
Barthel index, mean (SD)	64.1 (30.9)
Barthel index categories, n (%)	
Independent	16 (7.6)
Mild dependence	48 (22.9)
Moderate dependence	67 (31.9)
Severe dependence	49 (23.3)
Total dependence	30 (14.3)
Clinical Frailty Scale (CFS), mean (SD)	5.88 (1.28)
Frailty grade, n (%)	
Robust	25 (11.9)
Mild-moderate frailty	110 (52.4)
Severe-very severe frailty	75 (35.7)
Frailty (CFS ≥ 5), n (%)	185 (88.1)
Charlson comorbidity index, mean (SD)	3.64 (1.90)
Comorbidity level, n (%)	
None	24 (11.4)
Low	38 (18.1)
High	148 (70.5)
MNA-SF score, mean (SD)	8.85 (2.60)
Nutritional status, n (%)	
Malnourished	56 (26.9)
At risk of malnutrition	118 (56.7)
Normal	34 (16.3)
Polypharmacy, n (%)	194 (91.4)
Electrocardiogram, n (%)	
Sinus rhythm	64 (30.5)
Atrial fibrillation	127 (60.5)
Pacemaker rhythm	19 (9.0)
Hypertension, n (%)	182 (86.7)
Diabetes mellitus, n (%)	64 (30.5)
Chronic kidney disease, n (%)	96 (45.7)
Ischemic heart disease, n (%)	44 (21.0)
Atrial fibrillation, n (%)	142 (67.9)
Pulmonary hypertension, n (%)	108 (52.2)
LVEF category, n (%)	
Reduced	15 (7.2)
Mildly reduced	26 (12.6)
Preserved	166 (80.2)
Hemoglobin, g/dL, mean (SD)	11.74 (2.01)
Creatinine, mg/dL, mean (SD)	1.30 (0.72)
eGFR (CKD-EPI), mL/min/1.73 m^2^, mean (SD)	47.5 (18.2)
Sodium, mEq/L, mean (SD)	138.46 (4.72)
Potassium, mEq/L, mean (SD)	4.64 (2.80)
NT-proBNP, pg/mL, median (P25–P75)	5906 (3070–11,599)
CA125, U/mL, median (P25–P75)	46.7 (24.1–108.0)
Pleural effusion on chest X-ray, n (%)	166 (79.0)
ACE inhibitor, n (%)	92 (43.8)
Mineralocorticoid receptor antagonist, n (%)	44 (21.0)
Beta-blocker, n (%)	98 (46.7)
Sacubitril/valsartan, n (%)	4 (1.9)
SGLT2 inhibitor, n (%)	36 (17.1)
Inferior vena cava diameter, mm, mean (SD)	22.5 (4.2)
Inferior vena cava ≥ 21 mm, n (%)	137 (65.6)
Peripheral edema, n (%)	
None	44 (21.0)
Ankle edema	45 (21.4)
Below-knee edema	72 (34.3)
Above-knee edema	49 (23.3)
Jugular venous distension, n (%)	112 (53.3)
Hepatomegaly, n (%)	40 (19.1)
Orthopnea, n (%)	106 (50.5)
Composite Congestion Score (CCS), median (P25–P75)	1.67 (1.00–2.67)
Congestion grade, n (%)	
No congestion (CCS < 1)	48 (22.9)
Mild congestion (CCS 1–2)	138 (65.7)
Severe congestion (CCS = 3)	24 (11.4)
Obesity (BMI ≥ 30 kg/m^2^), n (%)	40 (19.3)

^a^ Values are presented as mean (SD) for normally distributed continuous variables, median (P25–P75) otherwise, and n (%) for categorical variables. Abbreviations: BMI, body mass index; CA125, carbohydrate antigen 125; CCS, composite congestion score; CFS, Clinical Frailty Scale; CKD-EPI, Chronic Kidney Disease Epidemiology Collaboration equation; eGFR, estimated glomerular filtration rate; LVEF, left ventricular ejection fraction; MNA-SF, Mini Nutritional Assessment–Short Form; NT-proBNP, N-terminal pro–B-type natriuretic peptide; SGLT2, sodium–glucose cotransporter 2.

**Table 2:** Baseline characteristics according to CA125 tertiles.

Variable ᵃ	Tertile 1 (n = 63)	Tertile 2 (n = 63)	Tertile 3 (n = 62)	*p*-Value
Age, years, mean (SD)	89.3 (4.9)	89.7 (5.4)	89.8 (5.1)	0.811
Female sex, n (%)	43 (68.3)	44 (69.8)	50 (80.6)	0.238
BMI, kg/m^2^, mean (SD)	27.3 (5.7)	24.8 (4.7)	25.0 (5.1)	**0.015**
Living situation: Nursing home, n (%)	21 (33.3)	20 (31.7)	24 (38.7)	0.693
Barthel index, mean (SD)	69.9 (29.3)	69.0 (27.4)	63.8 (30.7)	0.461
Barthel index categories, n (%)				0.887
Independent	5 (7.9)	6 (9.5)	5 (8.1)	
Mild dependence	19 (30.2)	15 (23.8)	12 (19.4)	
Moderate dependence	19 (30.2)	24 (38.1)	21 (33.9)	
Severe dependence	13 (20.6)	13 (20.6)	15 (24.2)	
Total dependence	7 (11.1)	5 (7.9)	9 (14.5)	
Clinical Frailty Scale (CFS), mean (SD)	5.70 (1.25)	5.71 (1.29)	5.95 (1.35)	0.476
Frailty grade, n (%)				0.466
Robust	8 (12.7)	9 (14.3)	8 (12.9)	
Mild–moderate frailty	34 (54.0)	40 (63.5)	31 (50.0)	
Severe–very severe frailty	21 (33.3)	14 (22.2)	23 (37.1)	
Frailty (CFS ≥ 5), n (%)	55 (87.3)	54 (85.7)	54 (87.1)	0.960
Charlson comorbidity index, mean (SD)	3.32 (1.75)	3.75 (1.84)	3.61 (1.85)	0.400
Comorbidity level, n (%)				0.707
None	9 (14.3)	6 (9.5)	9 (14.5)	
Low	14 (22.2)	13 (20.6)	9 (14.5)	
High	40 (63.5)	44 (69.8)	44 (71.0)	
MNA-SF score, mean (SD)	9.52 (2.51)	8.94 (2.36)	8.92 (2.51)	0.296
Nutritional status, n (%)				0.725
Malnourished	12 (19.0)	14 (22.2)	16 (25.8)	
At risk of malnutrition	37 (58.7)	40 (63.5)	36 (58.1)	
Normal	14 (22.2)	9 (14.3)	10 (16.1)	
Polypharmacy, n (%)	60 (95.2)	56 (88.9)	59 (95.2)	0.274
Electrocardiogram rhythm, n (%)				0.216
Sinus rhythm	25 (39.7)	18 (28.6)	14 (22.6)	
Atrial fibrillation	31 (49.2)	40 (63.5)	43 (69.4)	
Pacemaker rhythm	7 (11.1)	5 (7.9)	5 (8.1)	
Hypertension, n (%)	55 (87.3)	53 (84.1)	56 (90.3)	0.583
Diabetes mellitus, n (%)	21 (33.3)	22 (34.9)	16 (25.8)	0.503
Chronic kidney disease, n (%)	25 (39.7)	29 (46.0)	29 (46.8)	0.679
Ischemic heart disease, n (%)	11 (17.5)	14 (22.2)	16 (25.8)	0.526
Atrial fibrillation, n (%)	37 (58.7)	42 (67.7)	48 (77.4)	0.082
Pulmonary hypertension, n (%)	27 (45.0)	32 (50.8)	38 (61.3)	0.188
LVEF category, n (%)				0.157
Reduced	3 (5.0)	4 (6.3)	8 (12.9)	
Mildly reduced	4 (6.7)	10 (15.9)	10 (16.1)	
Preserved	53 (88.3)	49 (77.8)	44 (71.0)	
Hemoglobin, g/dL, mean (SD)	11.78 (1.89)	11.69 (1.88)	11.79 (2.11)	0.953
Creatinine, mg/dL, mean (SD)	1.18 (0.50)	1.21 (0.48)	1.40 (1.07)	0.183
eGFR (CKD-EPI), mL/min/1.73 m^2^, mean (SD)	50.6 (17.8)	49.6 (19.8)	48.0 (20.1)	0.747
Sodium, mEq/L, mean (SD)	138.4 (3.8)	138.4 (4.8)	138.2 (5.1)	0.941
Potassium, mEq/L, mean (SD)	4.97 (5.0)	4.36 (0.55)	4.58 (0.59)	0.501
NT-proBNP, pg/mL, median (P25–P75)	3717 (1907–7548)	6974 (4000–11,076)	5954 (3467–16,165)	**<0.001**
CA125, U/mL, median (P25–P75)	15.7 (7.9–22.9)	43.0 (35.5–56.4)	137.0 (96.6–207.8)	—
ACE inhibitor, n (%)	29 (46.0)	29 (46.0)	28 (45.2)	0.994
Mineralocorticoid receptor antagonist, n (%)	14 (22.2)	10 (15.9)	14 (22.6)	0.574
Beta-blocker, n (%)	29 (46.0)	28 (44.4)	30 (48.4)	0.906
Sacubitril/valsartan, n (%)	3 (4.8)	0	1 (1.6)	0.113
SGLT2 inhibitor, n (%)	13 (20.6)	13 (20.6)	8 (12.9)	0.432
Heart failure readmission, n (%)	33 (52.4)	32 (50.8)	39 (62.9)	0.335
Mortality, n (%)	19 (30.2)	21 (33.3)	30 (48.4)	0.080
Composite outcome: heart failure readmission or mortality, n (%)	36 (57.1)	38 (60.3)	46 (74.2)	0.109

ᵃ Values are presented as mean (SD) for normally distributed continuous variables, median (P25–P75) otherwise, and n (%) for categorical variables. Statistically significant results (*p* < 0.05) are shown in bold. Abbreviations: BMI, body mass index; CA125, carbohydrate antigen 125; CFS, Clinical Frailty Scale; CKD-EPI, Chronic Kidney Disease Epidemiology Collaboration equation; eGFR, estimated glomerular filtration rate; LVEF, left ventricular ejection fraction; MNA-SF, Mini Nutritional Assessment–Short Form; NT-proBNP, N-terminal pro–B-type natriuretic peptide; SGLT2, sodium–glucose cotransporter 2.

**Table 3:** Association of CA125 levels with all-cause mortality and with the composite of all-cause mortality or heart failure hospital readmissions.

CA125 (U/mL)	Category	All-Cause Mortality, HR (95% CI)	*p*-Value	Composite Outcome, HR (95% CI)	*p*-Value
<28.2	T1	1.00 (reference)	—	1.00 (reference)	—
28.2–75.4	T2	1.17 (0.63–2.18)	0.618	1.09 (0.69–1.73)	0.699
>75.4	T3	1.89 (1.06–3.35)	**0.031**	1.60 (1.03–2.48)	**0.035**
≥100	—	1.88 (1.15–3.09)	**0.012**	1.54 (1.04–2.29)	**0.031**

Cox regression analysis showing the association between CA125 levels and all-cause mortality and the composite endpoint. Statistically significant results (*p* < 0.05) are shown in bold. Abbreviations: CA125, carbohydrate antigen 125; CI, confidence interval; HR, hazard ratio.

## Data Availability

The data presented in this study are available on request from the corresponding author due to privacy and ethical restrictions.

## Abbreviations

The following abbreviations are used in this manuscript:

ACEI	Angiotensin-converting enzyme inhibitor
AF	Atrial fibrillation
AHF	Acute heart failure
AUC	Area under the curve
BI	Barthel Index
BMI	Body mass index
CA125	Carbohydrate antigen 125
CCS	Composite congestion score
CFS	Clinical Frailty Scale
CGA	Comprehensive geriatric assessment
CI	Confidence interval
CKD	Chronic kidney disease
CKD-EPI	Chronic Kidney Disease Epidemiology Collaboration (equation)
CRP	C-reactive protein
DM	Diabetes mellitus
ECG	Electrocardiogram
EF	Ejection fraction
eGFR	Estimated glomerular filtration rate
HBP	High blood pressure
HF	Heart failure
HFpEF	Heart failure with preserved ejection fraction
HR	Hazard ratio
HTN	Hypertension
IQR	Interquartile range
LVEF	Left ventricular ejection fraction
MNA-SF	Mini Nutritional Assessment—Short Form
MRA	Mineralocorticoid receptor antagonist
NT-proBNP	N-terminal pro-B-type natriuretic peptide
OR	Odds ratio
P25–P75	25th–75th percentiles
PH	Pulmonary hypertension
ROC	Receiver operating characteristic
SD	Standard deviation
SGLT2i	Sodium–glucose cotransporter 2 inhibitor
SPSS	Statistical Package for the Social Sciences
VIDAS	bioMérieux immunoassay platform
